# The Late Captain H. S. Tunnard

**Published:** 1917-12-15

**Authors:** 


### AN ORGANISER AND ADMINISTRATOR.

### The Late Captain H. S. Tunnard.

All associated with King's College Hospital will miss Captain Tunnard and mourn his loss. Captain Tunnard was taken suddenly ill after the dedication of the memorial reredos to the late Matron, Miss Katharine Monk, on November 1, and his death at the early age of sixty-one came with a suddenness, which added, if possible, to the keenness of the grief arising out of it.

Appointed Secretary in 1901, Captain Tunnard came to his new work on retiring from the Army, and although he had had no special training for it, his administrative gifts, great abilities, and personal devotion to his duties (he was a master of detail and an untiring worker) soon caused his advent to be welcomed by all, who had the best interests of King's College Hospital at heart. The hospital had before it enormous difficulties, including the selection of a new site, the erection thereon of a modern, up-to-date hospital with the many adjuncts essential to the efficient working of its medical school, the raising of a great Building Fund, and the removal of the patients to Denmark Hill. A man of less ability and experience who had not had the advantage of a military training—Captain Tunnard was an officer of the late Royal Inniskilling Fusiliers, 27th Foot—might well have been appalled by the task which fell to his lot within two years of his appointment as Secretary. But he soon mastered the necessary details and got the work at Portugal Street well in hand before the question of removal to South London became acute.

He had the good fortune to be associated with the late Sister-Matron, Miss Katharine Monk, who, clever woman that she was, speedily recognised and appreciated Captain Tunnard's qualities, and formed a high regard for his organising ability. They both took the keenest interest in the new schemes, and their zealous co-operation together had much to do with the notable success which was ultimately achieved. Once the present site abutting on Ruskin Park was secured, under the leadership and inspiration of the late Sister-Matron, the work as it grew was mastered and organised, so that the joint labours of these two officers smoothed the way for all those engaged in the work, and resulted in a perfected system attended by triumphant results. During the whole period from the acquirement of the site to the completion of the buildings and their equipment for occupation, Captain Tunnard allowed no thread of any value to the scheme to escape him, and he maintained an unwearied enthusiasm and watchfulness throughout. Like Sister Katharine, he too was a great organiser, and in the equipment of the new hospital at Denmark Hill Miss Ray, the present Sister-Matron, proved a competent colleague, ever ready to direct and suggest. The most difficult of all difficulties in such a removal—a matter of great economical importance—was the survey and utilisation of as much of the equipment of the old hospital as possible. In this connection Captain Tunnard devised and scheduled a time-table of the evacuation from the old hospital to the occupation of the new one. Labour troubles added greatly to the difficulties which had to be faced, but so perfect was his control and untiring his energy, that the change was effected expeditiously, and the old and new equipment arrived in agreement with the time-table, and was delivered to the appointed room or place as specified on the schedule, in perfect order and without the slightest confusion. The original selection of Captain Tunnard was a wise step on the part of the committee, and it is but just to say that the hospital at Denmark Hill will always be Captain Tunnard's record of devoted service, a labour of love and life-long interest. He had no axes to grind, no personal ambitions, and indeed, his chief characteristics were his faculty of calling for admiration for work accomplished by his colleagues and associates, his unfaltering loyalty to his superiors, and his whole-hearted devotion to the interests of King's College Hospital, where he will be missed and greatly remembered as the years pass.

The hospital world knew very little of Captain Tunnard, who was of a retiring disposition. He was a man of admirable judgment and diplomacy, and these, coupled with his military training, made him an excellent chief of the heads of the departments which constitute a hospital, where everything under his *régime* worked smoothly and without a hitch. His life-work proves him to have been a man of the precise type, which the more important hospitals of this country require at the head of their administration. Captain Tunnard, indeed, possessed just the qualities, including education and training for high office, that hospital committees discover it is so difficult to meet with, when the need arises.

